# Enhancement of critical-sized bone defect regeneration using UiO-66 nanomaterial in rabbit femurs

**DOI:** 10.1186/s12917-022-03347-9

**Published:** 2022-07-05

**Authors:** Ahmed Abdelrahiem Sadek, Mahmoud Abd-Elkareem, Hani Nasser Abdelhamid, Samia Moustafa, Kamal Hussein

**Affiliations:** 1 Department of Surgery, Anesthesiology, and Radiology, Faculty of Veterinary Medicine, Assiut University, Assiut, Egypt; 2 Department of Cell and Histology, Faculty of Veterinary Medicine, Assiut University, Assiut, Egypt; 3 Advanced Multifunctional Materials Laboratory, Department of Chemistry, Faculty of Science, Assiut University, Assiut, Egypt; 4 Proteomics Laboratory for Clinical Research and Materials Science, Department of Chemistry, Faculty of Science, Assiut, Egypt; 5 Nanotechnology Research Centre (NTRC), The British University in Egypt, El-Shorouk City, Suez Desert Road, P.O. Box 43, Cairo, 11837 Egypt

**Keywords:** UiO-66, Critical-sized bone defect, Bone healing, Regeneration

## Abstract

**Background:**

Repair of large-sized bone defects is a challengeable obstacle in orthopedics and evoked the demand for the development of biomaterials that could induce bone repair in such defects. Recently, UiO-66 has emerged as an attractive metal–organic framework (MOF) nanostructure that is incorporated in biomedical applications due to its biocompatibility, porosity, and stability. In addition, its osteogenic properties have earned a great interest as a promising field of research. Thus, the UiO-66 was prepared in this study and assessed for its potential to stimulate and support osteogenesis in vitro and in vivo in a rabbit femoral condyle defect model. The nanomaterial was fabricated and characterized using x-ray diffraction (XRD) and transmission electron microscopy (TEM). Afterward, in vitro cytotoxicity and hemolysis assays were performed to investigate UiO-66 biocompatibility. Furthermore, the material in vitro capability to upregulate osteoblast marker genes was assessed using qPCR. Next, the in vivo new bone formation potential of the UiO-66 nanomaterial was evaluated after induction of bone defects in rabbit femoral condyles. These defects were left empty or filled with UiO-66 nanomaterial and monitored at weeks 4, 8, and 12 after bone defect induction using x-ray, computed tomography (CT), histological examinations, and qPCR analysis of osteocalcin (OC) and osteopontin (OP) expressions.

**Results:**

The designed UiO-66 nanomaterial showed excellent cytocompatibility and hemocompatibility and stimulated the in vitro osteoblast functions. The in vivo osteogenesis was enhanced in the UiO-66 treated group compared to the control group, whereas evidence of healing of the treated bone defects was observed grossly and histologically. Interestingly, UiO-66 implanted defects displayed a significant osteoid tissue and collagen deposition compared to control defects. Moreover, the UiO-66 nanomaterial demonstrated the potential to upregulate OC and OP in vivo.

**Conclusions:**

The UiO-66 nanomaterial implantation possesses a stimulatory impact on the healing process of critical-sized bone defects indicating that UiO-66 is a promising biomaterial for application in bone tissue engineering.

**Supplementary Information:**

The online version contains supplementary material available at 10.1186/s12917-022-03347-9.

## Background

Bone healing is a coordinating process of synchronized stages aiming to form a new functional bone tissue with normal architecture rather than scar tissue. Unfortunately, in extensive bone damage related to high energy trauma, bone infection debridement, bone cancer resection, blast injuries, and skeletal deformities, the bone repair is impaired [1, 2]. Osseous defects that fail to regenerate spontaneously with de novo bone tissue because their size surpasses the intrinsic healing potential are defined as critical-sized bone defects [2]. These critical-sized bone defects are regarded as an important health issue with economic significance in veterinary practice [3, 4].

Traditional clinical strategies, including autogenic and allogenic bone grafts, are used to repair the critical-sized bone defect [1, 3, 5]. Autogenous bone grafts are the gold standard treatment used for this purpose due to their osteoconductive, osteoinductive, and osteogenic properties. However, their clinical use has been confined due to limited sources of autogenous bone tissue, bleeding, donor site morbidity, and prolonged grafting time [6–8]. Allogenic bone graft is an alternative treatment that commonly associated with the risk of immunological rejection, the transmission of infectious diseases, and limited incorporation into host bone [8, 9]. Consequently, bone tissue engineering has attracted significant attention as a potential alternative solution to overcome these limitations and fulfill the clinical need [5, 8, 9]. Bone tissue engineering consists of a combination of scaffolds, seeded cells, and/or growth factors. Scaffolds are considered a crucial component in bone tissue engineering as they act as a platform for cellular interaction and provide a suitable microenvironment with a structural framework and mechanical cues for the newly formed osseous tissue [1, 5, 9]. Consequently, the scaffold material should be biocompatible, biodegradable, and mechanically stable, supporting biological activities such as cell attachment, migration, and growth. Various biomaterials have been used in bone tissue engineering, such as ceramics, metals, and polymers; however, these materials have the disadvantages of poor osteoinductivity, weak bioactivity, and low biodegradation [10].

Metal–organic frameworks (MOFs)-based materials have been extensively explored as promising candidates for biomedical applications such as biosensing, bioimaging, drug delivery, gene delivery, and photodynamic therapy [11, 12]. These MOFs are composed of coordination between metal ions/clusters and organic ligands/linkers, and they have the advantages of large specific surface area, high porosity, high surface acidity and basicity, and excellent biocompatibility and biodegradability [10, 13–20]. Furthermore, the nanoscale MOFs in recent years have gained considerable attention in tissue regeneration [10]. Several MOFs have been reported to incorporate in the fabrication of scaffolds or implants used in bone tissue engineering such as zeolitic imidazolate framework-8 [14], iron-based MOFs [21], and zinc-based MOFs [22]. The nanosized MOFs have been reported to create a favorable extracellular environment that stimulates and supports mesenchymal stem cells (MSCs) adhesion, proliferation, and differentiation [23]. In addition, it has been suggested that incorporation of nanoscale MOFs to the designed scaffolds improves the adherence and interactions of MSCs owing to modification of the functional groups, porosity, roughness, and hydrophilicity of scaffolds [10].

Among the various MOF biomaterials, the University Institute of Oslo-66 (UiO-66) is porous zirconium (Zr) MOF that has the advantages of low toxicity, porosity, biocompatibility, and excellent chemical and thermal tenability [24–27]. Thus, UiO-66 has been applied in various applications, including energy [28, 29] and biomedical fields such as drug delivery [24, 30] and photothermal therapy [31].

Zr-containing biomaterials have been used extensively in orthopedic and dental implants. Zr has the potential to induce osteogenesis via stimulation of osteoblast attachment, proliferation, and differentiation, enormously increasing the expression of the osteogenic genes and enhancing the mineralization process [32]. In the present study, we hypothesized that UiO-66 nanomaterial has a positive boosting osteogenic effect in the regeneration of critical-sized bone defects. Hence, the current study aimed to evaluate the UiO-66 scaffold's biocompatibility and its potential to induce and support osteogenesis in vitro and in vivo in a rabbit femoral condyle model.

## Results

### Characterization of UiO-66 nanomaterial

**Fig. 1:**
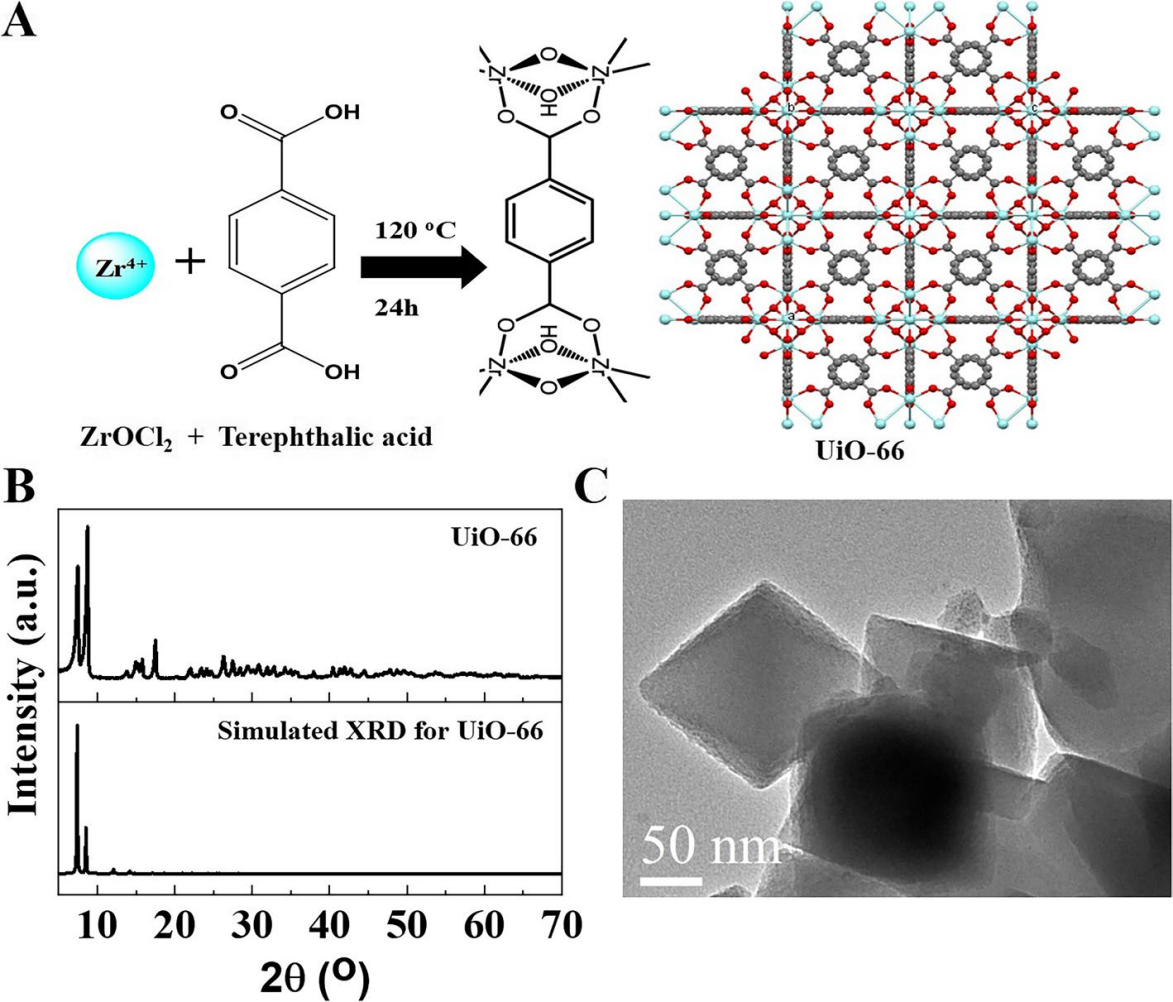
Synthesis and characterization of UiO-66 nanomaterial. **A** Schematic representation for the synthesis of UiO-66. **B** The XRD pattern of UiO-66, and **C** TEM image of UiO-66. TEM image is reprinted with permission from Reference 13

UiO-66 was successfully synthesized, as shown in Fig. 1A. The solvothermal synthesis of UiO-66 resulted in a white precipitate. XRD patterns of the synthesized and simulated pattern of UiO-66 were matched very well (Fig. 1B). TEM image of UiO-66 showed a crystal with a particle size of 170 nm (Fig. 1C).

### Indirect contact cytotoxicity assay

**Fig. 2:**
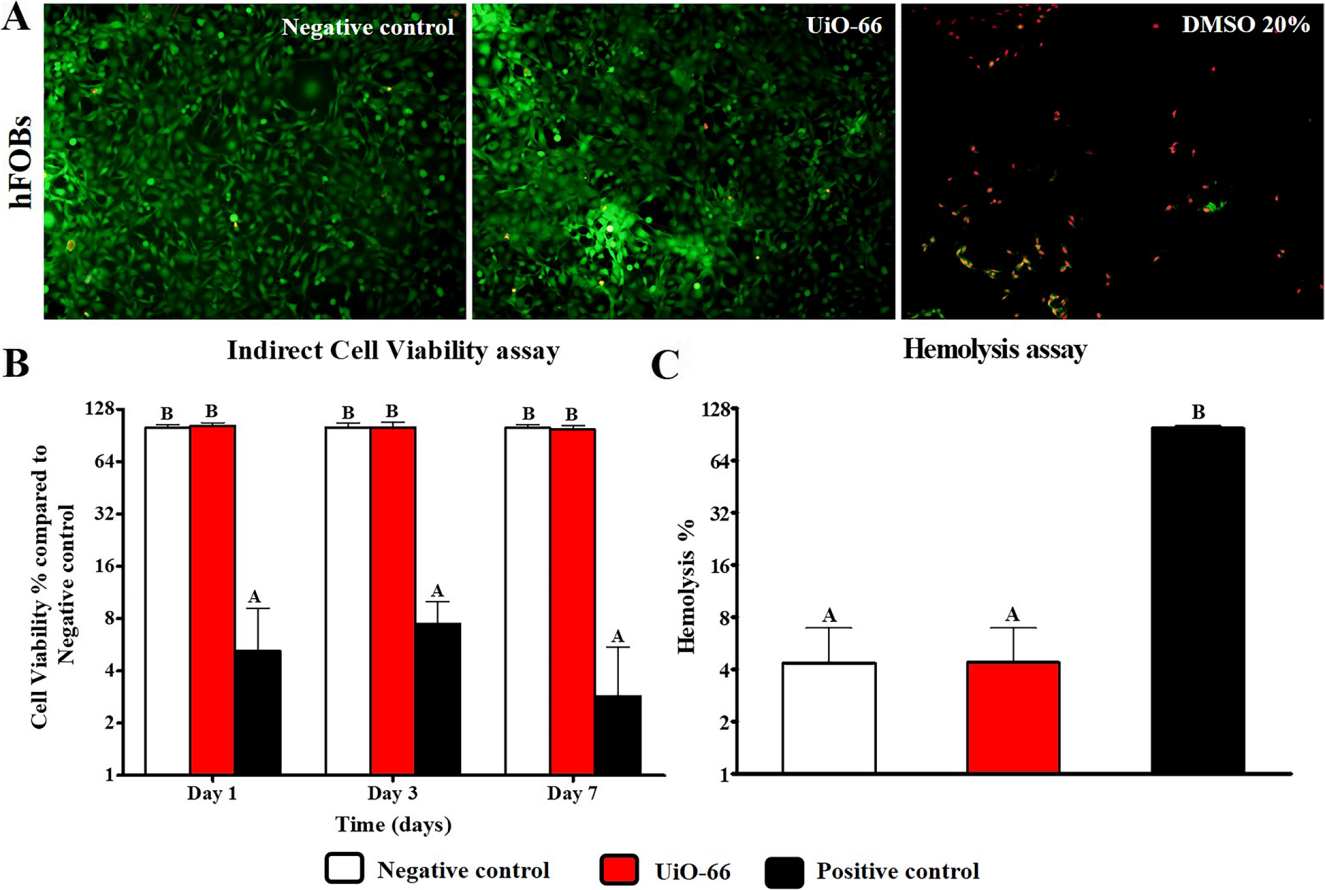
In vitro assessment of UiO-66 biocompatibility. **A** Cell viability using Live/Dead assay for hFOBs seeded on UiO-66 nanomaterial for 7 days. Green cells represent live cells and red color indicates dead cells (Scale = 100 µm, Magnification = 10x). **B** MTT cytotoxicity assay for viability evaluation of hFOB cells cultured using extracts of UiO-66 material for 1, 3, and 7 days compared to the negative control. Error bars represent means ± SD (*n* = 8). **C** Hemolysis assay using extracts of UiO-66 nanomaterial compared to the negative control. Error bars represent means ± SD (*n* = 3). Bars with the same letter represent insignificance values (one-way ANOVA followed by Tukey's HSD post hoc test)

The preconditioned media prepared using UiO-66 nanomaterial samples showed few positive cells to ethidium bromide homodimer staining after 7 days of culture (Fig. 2A). The cell viability was measured using MTT assay through investigation of hFOB cells proliferation using extraction media prepared from different samples. It showed a non-significant level of cell growth and proliferation between hFOB cells cultured in DMEM (negative control) and that cultured using preconditioned extracts from UiO-66 nanomaterial after 1, 3, and 7 days of culture (Fig. 2B).

### Hemolysis assay

As shown in Fig. 2C, the UiO-66 nanomaterial showed a non-significant hemolysis rate (9.64% ± 4.58%) compared to the negative control group (4.35% ± 2.58%). This indicated that the designed nanomaterials do not have a hemolytic effect and have good blood compatibility.

### qPCR analysis

**Fig. 3:**
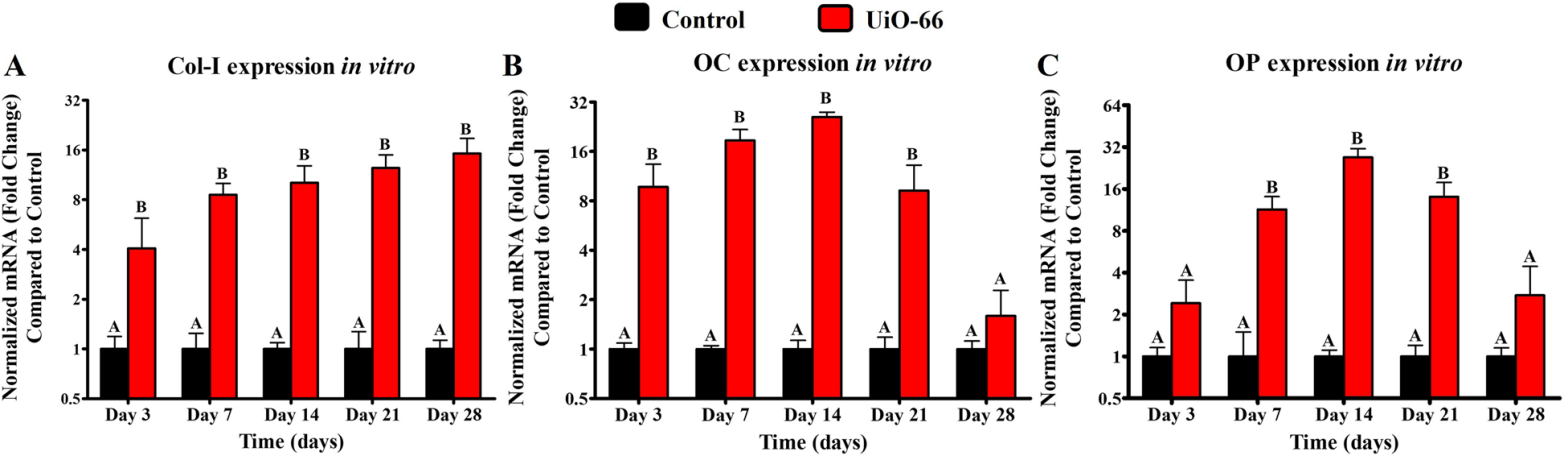
In vitro osteogenic potential of UiO-66. qPCR analysis for the expression level of collagen type-I (Col-I) (**A**), osteocalcin (OC) (**B**), and osteopontin (OP) (**C**) mRNA in negative control cells and on UiO-66 nanomaterial for 28 days, respectively. Error bars represent means ± SD; *n* = 3 for each group and time point. Bars with the same letter represent insignificance values (one-way ANOVA followed by Tukey's HSD post hoc test)

The expression of Col-I was significantly higher in cells cultured on Uio-66 nanomaterial compared to that in the negative control group starting from day 3 till day 28 (Fig. 3A). However, cells cultured on Uio-66 showed significantly higher OC and OP expression levels than the negative control group at different times except on day 28 for OC expression (Fig. 3B) and both day 3 and day 28 for OP expression (Fig. 3C).

### Clinical investigation

**Fig. 4:**
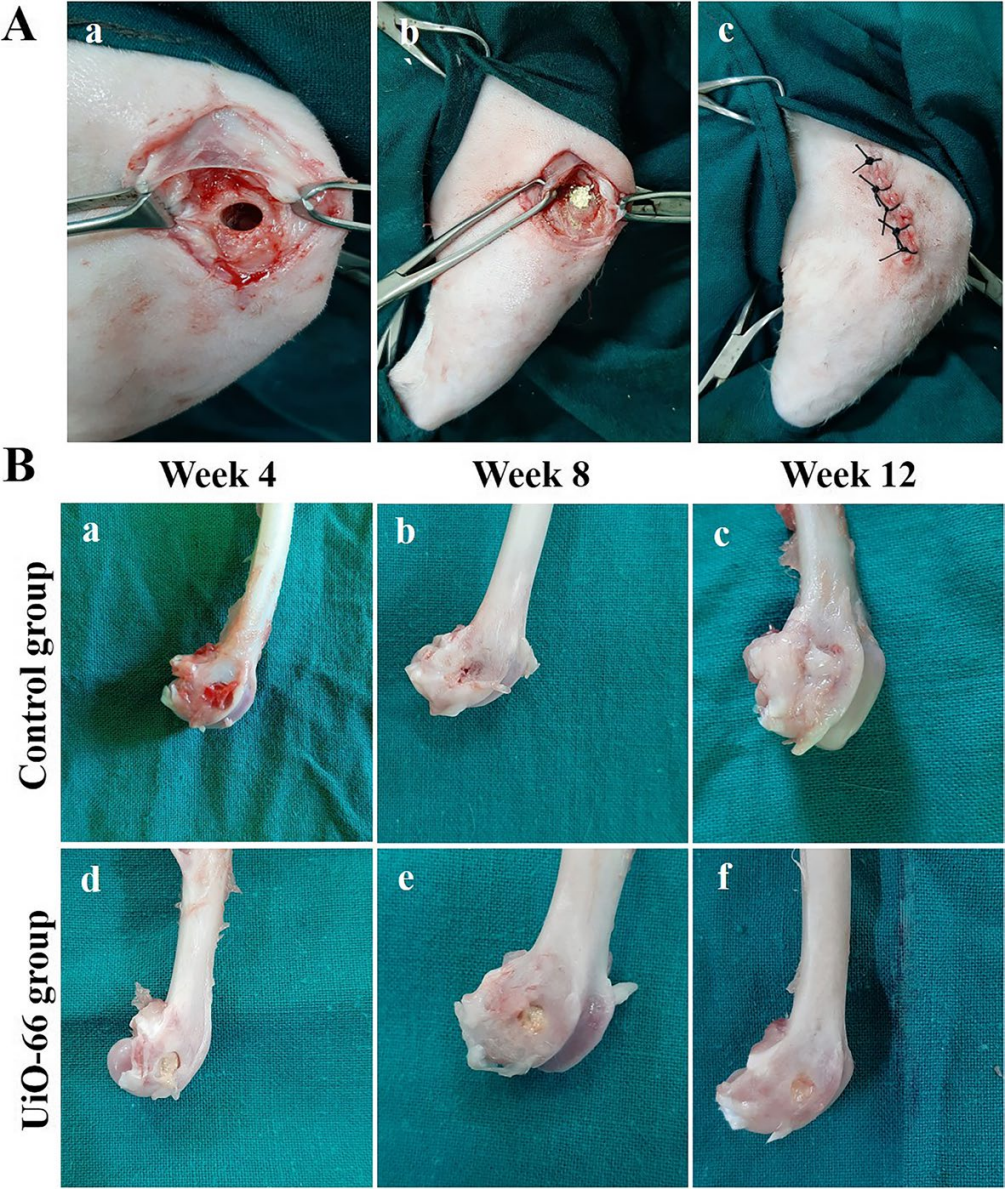
Gross appearance of the critical-sized bone defects. (A) Establishment of critical sized bone defect (Ø 5 × 10 mm) in rabbit femoral condyle (a), implantation of UiO-66 material (b), and wound closure (c). (B) Gross appearance of the rabbit femoral condyle bone defects of control (a-c) and UiO-66 implanted (d-f) groups at different evaluation times

As displayed in Fig. 4, the critical-sized bone defects were established successfully. All animals survived during the study period without operative or postoperative complications. They could stand up and walk on the first day after the operation and returned to their daily activities, including eating, drinking, and grooming within 48 h after surgery. The skin wound healed without complications within 7–10 days after surgery for both groups.

### Radiographic evaluation

**Fig. 5:**
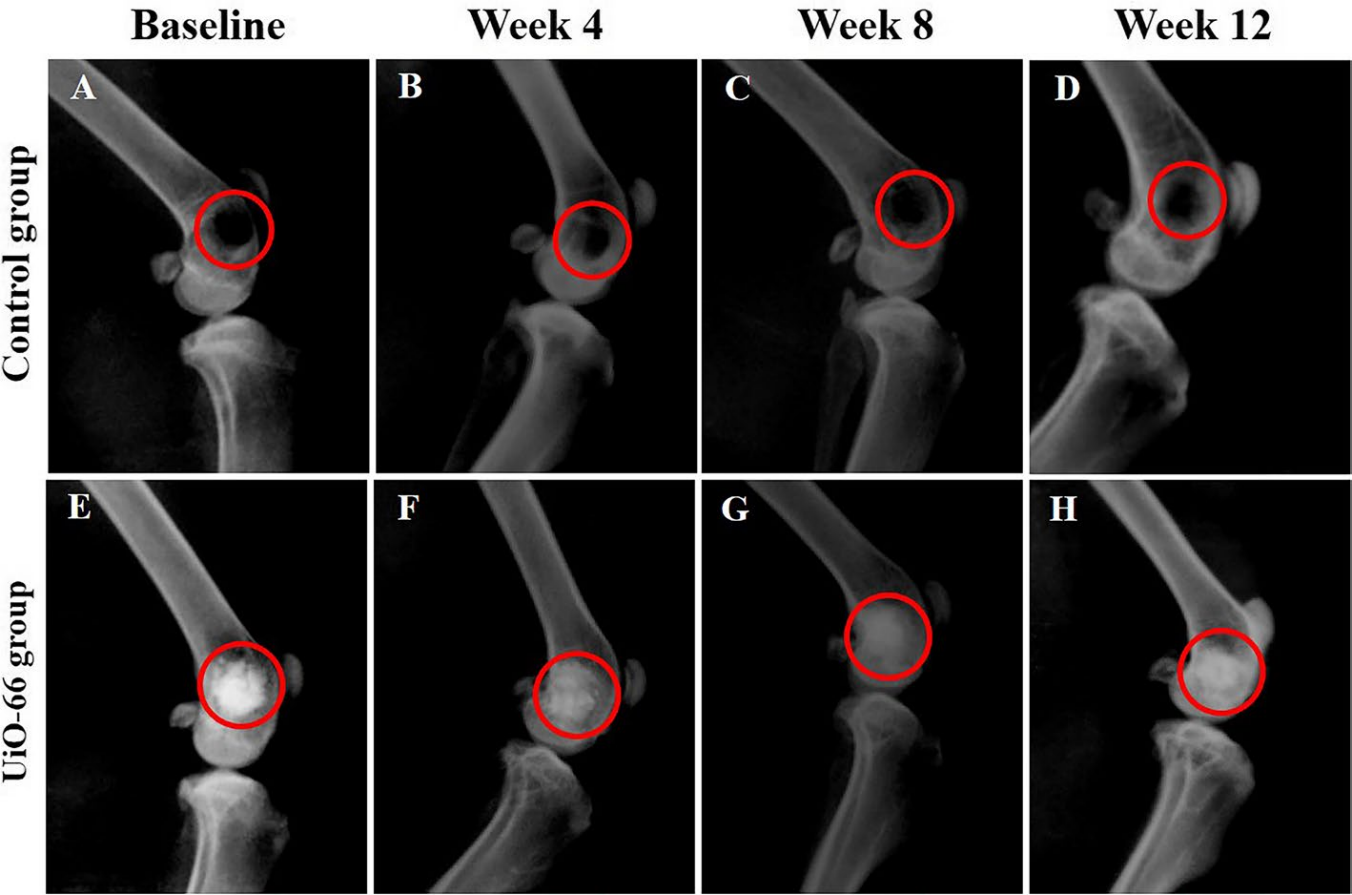
Radiographic evaluation of the defect site. Lateromedial radiographs of the defect site of control (**A**-**D**) and UiO-66 implanted (**E**–**H**) groups at different post-implantation times. Red circles: defect site

The immediate postoperative LM radiographs displayed well-demarcated radiolucent bone defects in the femoral condyles in the control group (Fig. 5A). UiO-66 implanted defects revealed highly radiopaque defects in the femoral condyles because of their higher radiographic density than the adjacent cancellous bone (Fig. 5E).

In the control group, the radiographs of the different evaluation times still showed a well-defined radiolucent bone defect (Fig. 5B-C). However, on week 12 after implantation, the bone defects displayed a tiny new bone formation at their margins (Fig. 5D).

In the UiO-66 treated group, the radiopacity at the bone defects decreased in a time-dependent manner (Fig. 5F-H). At week 4 after surgery, the defect margins were greatly indistinct. However, at weeks 8 and 12 post-implantation, the radiopacity decreased gradually, and the UiO-66 scaffolds were still clearly recognized in the bone defects.

### CT examination

**Fig. 6:**
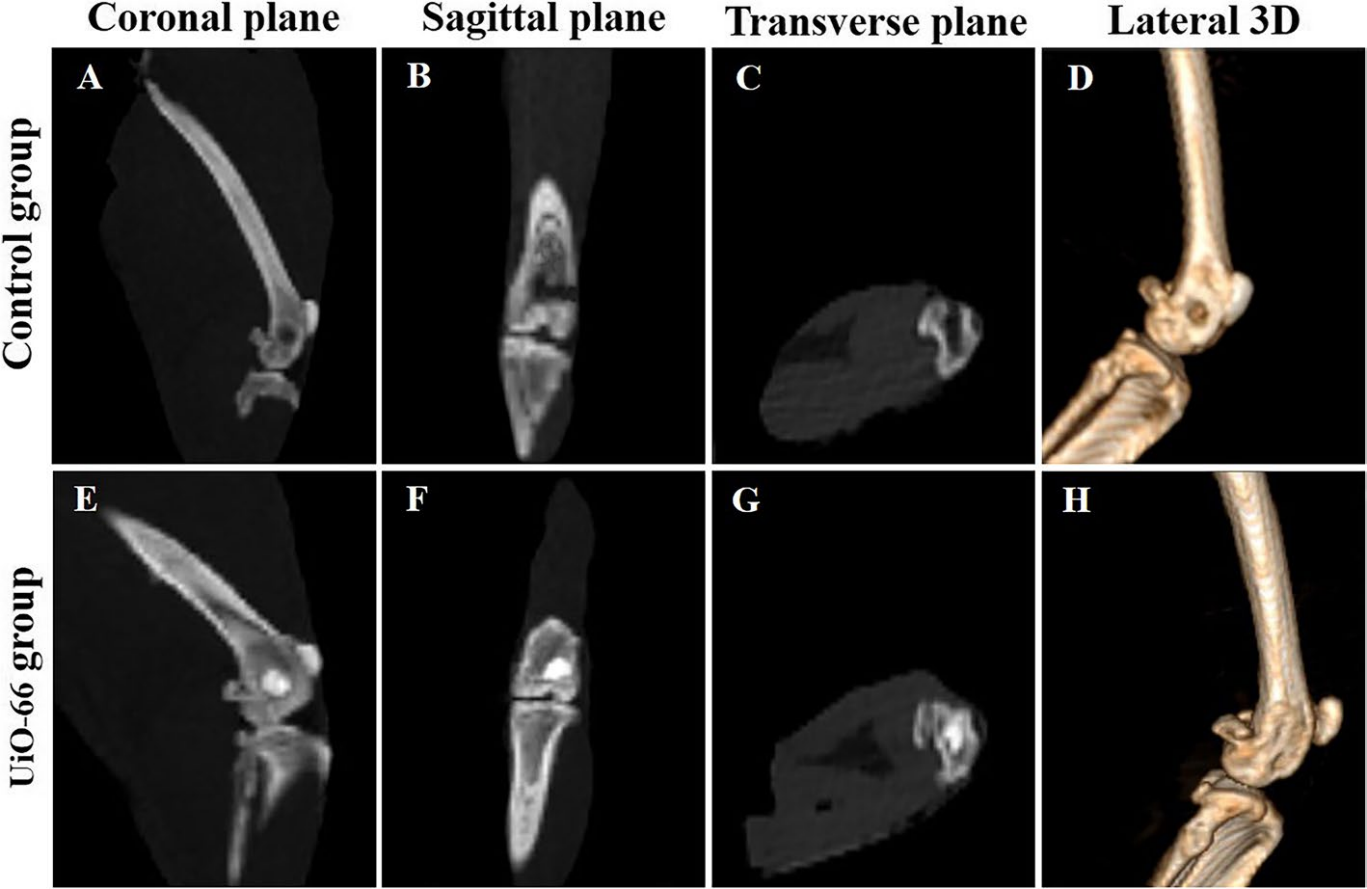
Computed tomography examination of the defect site. Coronal (**A**, **E**), sagittal (**B**, **F**), transverse (**C**, **G**) CT planes, and 3D CT images (**D**, **H**) of control (**A**-**D**) and UiO-66 implanted (**E**–**H**) groups at week 12 postoperatively

In the control group, bone defects appeared empty and clearly distinguished in the coronal, sagittal, and transverse planes (Fig. 6A-C). Conversely, UiO-66 implanted defects were appeared filled partially with newly formed bone with incomplete degradation to the scaffold in the different planes (Fig. 6E-G). Furthermore, in the lateral 3D-CT images, bone defects were clearly demarcated in the control group (Fig. 6D), while in the UiO-66 implanted group, the depth of the bone defect diminished with distinguishable defects’ margins (Fig. 6H).

### Gross evaluation of the femoral condyle bone defects

As shown in Fig. 4B, the specimens of different groups did not show any evidence of tissue infection in the adjacent soft tissues grossly. At week 4 after surgery, the margins of the defects were clearly demarcated in control and UiO-66 implanted groups. The bone defects of the control group were covered with blood clots and/or fatty tissue, while the UiO-66 implanted defects were covered with the yellow UiO-66 material. The UiO-66 scaffolds were well incorporated into bone defects and appeared integral to the surrounding host bone with undetectable interface between the scaffold and host bone.

On 8 and 12 weeks after surgical operation, the margins of the bone defects in the control group were still well-defined without any notable changes in the bone defects’ diameters, however; the UiO-66 implanted defects showed less distinguishable margins, decreased bone defects’ diameters, and partial repair of the bone defects with bridging smooth bone-like tissue connecting the rims of the bone defects.

### Histological assessment

Histological examination of the femoral condyle bone defects harvested on the postoperative 4, 8, and 12 weeks was carried out to investigate the osteogenic potential of UiO-66 nanomaterial to stimulate bone formation in the critical-sized bone defect model.

**Fig. 7:**
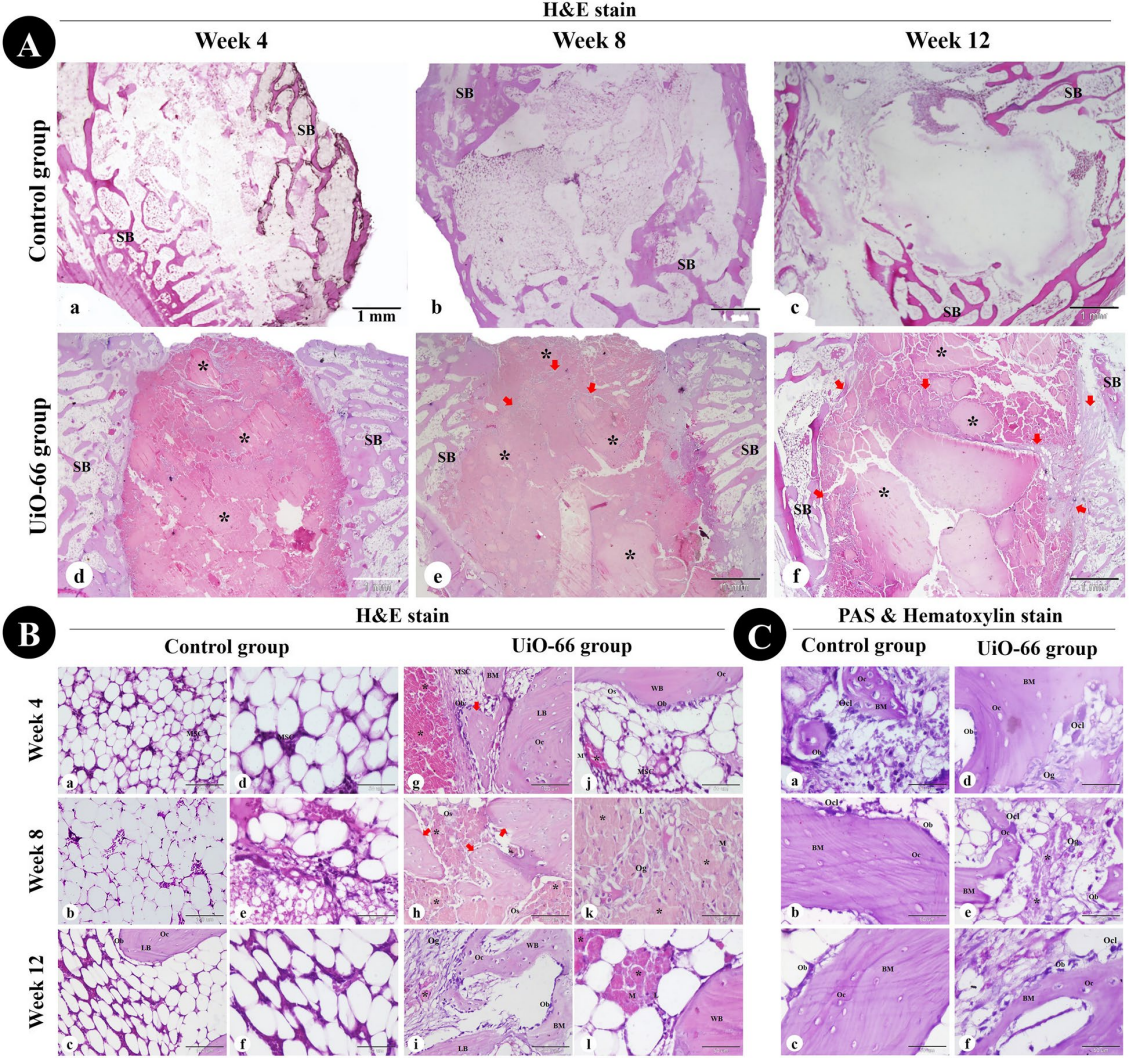
Histological assessment of critical-sized bone defect repair in rabbit femurs. (A) and (B) femoral condyle sections stained with Hematoxylin and Eosin from the control and UiO-66 implanted groups at weeks 4, 8, and 12 after surgery. (C) PAS & Hematoxylin- stained bone defect sites in control (a-c) and UiO-66 implanted (d-f) groups at different evaluation times. MSC: mesenchymal stem cell; Ob: osteoblast; Oc: osteocyte; Ocl: osteoclast; Og: osteogenic cells; Os: osteoid tissue; BM: bone matrix; SB: spongy bone; WB: woven bone; LB: lamellar bone; M: macrophage; L: lymphocyte; black asterisks: implanted UiO-66 nanomaterial; red arrows: newly formed bone. The scale bars in (A) = 1 mm, (B a-c,g-i) = 100 μm, and in (B d-f, j-l) and (C) = 50 μm

On week 4, the control group revealed empty defects or filled with fat cells and a few hematopoietic stem cells and MSCs (Fig. 7Aa & Ba,d). The UiO-66 implanted defects were occupied by dispersed UiO-66 nanomaterial separated by osteoid tissue, osteogenic cells, osteoblasts, osteoclasts, collagen fibers, and fibroblasts and surrounded by spongy bone trabeculae (Fig. 7Ad & Bg,j). Newly formed woven bone and some MSCs were observed in the periphery of the bone defects with a centripetal direction of osteo-regeneration (Fig. 7Ad & Bg). Furthermore, neovascularization, lymphoid aggregation, and macrophages were seen near the scaffold material in the implanted bone defect area (Fig. 7Bj).

On week 8, bone defects in the control group remained filled with fatty bone marrow (Fig. 7Ab & Bb,e). The UiO-66 implanted defects displayed more bone tissue formation and less scaffold material. The peripheral zones of bone defects were surrounded by the newly formed spongy bone trabeculae, whereas in the central zones, the remaining scaffold materials were surrounded and separated by osteoid tissue and newly formed woven bone trabeculae. Additionally, the implanted bone defect area still showed lymphoid infiltration and neovascularization near the scaffold material (Fig. 7Ae & Bh,k).

On week 12, bone repair in the control defects is still absent and cannot be observed (Fig. 7Ac & Bc,f). However, the UiO-66 implanted group revealed more anastomosed newly formed woven bone trabeculae centrally and connected to the peripheral newly formed lamellar bone. Neovascularization and inflammatory cell infiltration were still observed near the residual nanomaterial (Fig. 7Af & Bi,l).

PAS and hematoxylin-stained sections showed the absence of chondrocytes, while PAS-negative osteogenic cells, osteoblasts, osteocytes, and PAS-positive bone matrix were observed in the peripheral regions of all bone defects and the central areas of the implanted defects (Fig. 7C).

**Fig. 8:**
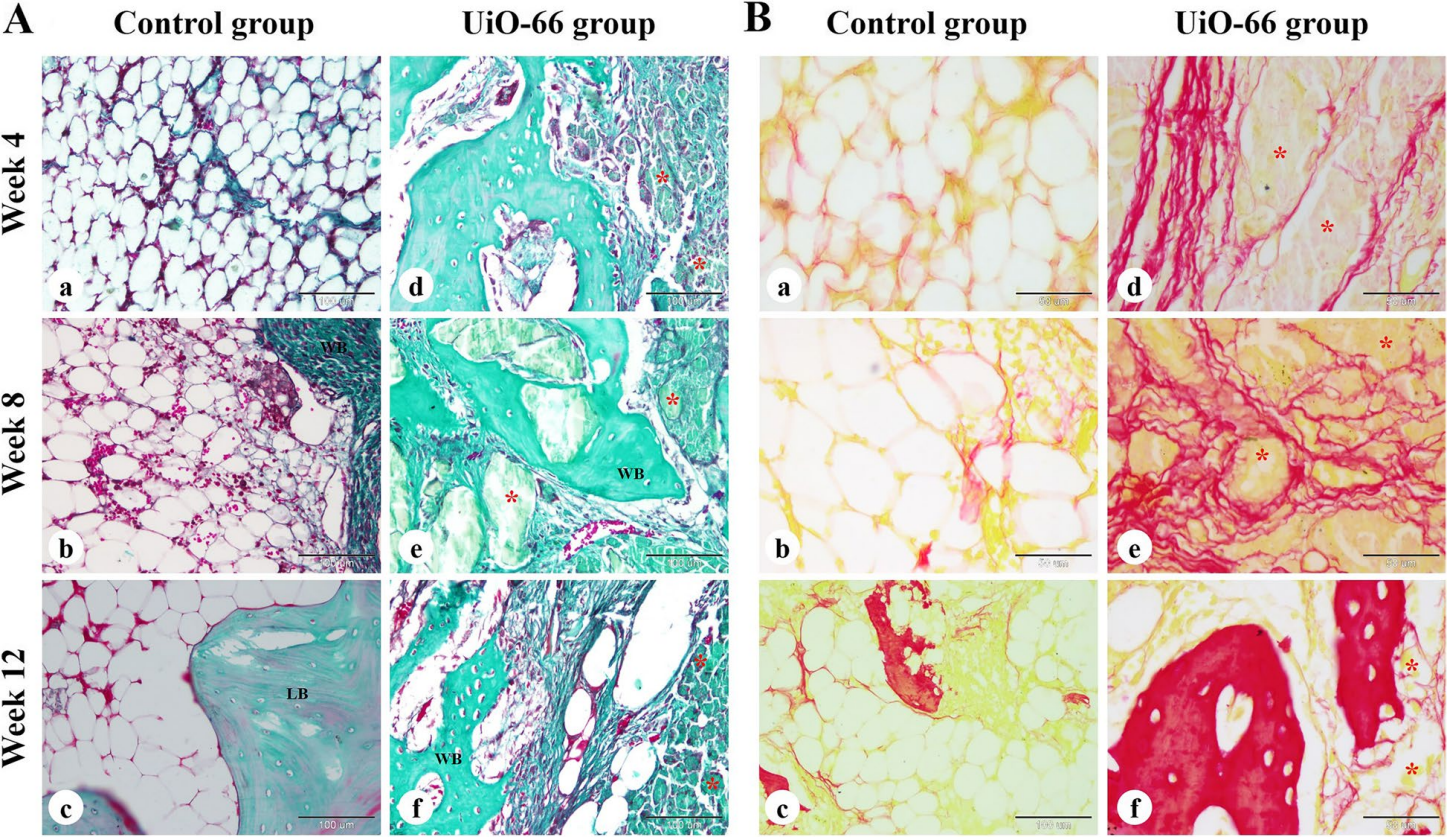
Histochemical examination of bone collagen. The repair site of the femoral condyle at weeks 4, 8, and 12 after surgery in control (a-c) and UiO-66 implanted (d-f) groups were stained with Crossmon's trichrome (A) and Sirius red (B) stains. WB: woven bone; LB: lamellar bone; red asterisks: implanted UiO-66 nanomaterial. The scale bars in Crossmon's trichrome stain panels = 100 μm and in Sirius red stain panels = 50 μm

The bone collagen deposition in the bone defects was further examined by Crossmon's trichrome and Sirius red staining (Fig. 8). The control group revealed the formation of newly formed bone containing mature collagen at the peripheral areas of the defect with unrepaired central areas. The UiO-66 implanted defects displayed mature bone collagen deposition at the newly formed bone matrix in the central and peripheral zones of the bone defects.

### Histological evaluation of the regenerated bone tissue

The woven immature bone was seen in the peripheral zone of the bone defects in control and UiO-66 implanted defects at different post-implantation times with a higher amount observed in the central zone of the UiO-66 implanted defects only. On the other hand, the mature lamellar bone was observed only in the peripheral region of the bone defects in both groups at weeks 8 and 12.

### Histomorphometric analysis

**Fig. 9:**
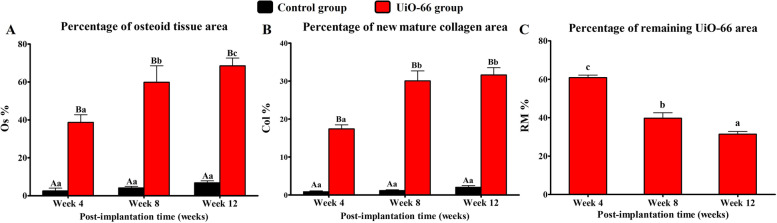
In vivo histomorphometric analysis. The osteoid tissue area (**A**), new mature collagen area (**B**), and remaining material area (**C**) percentages to the total defect area in control and UiO-66 implanted bone defects at various postoperative evaluation times. Error bars represent means ± SD; *n* = 3 for each group and time point. Bars with the same letter represent values that are not significantly different. A, B, and C: significance between groups; a, b, and c: significance between time points within the same group

As shown in Fig. 9, the UiO-66 implanted group at weeks 4, 8, and 12 displayed a significant increase in Os% (38.69 ± 4.03%, 59.90 ± 8.68%, and 68.52 ± 4.11%, respectively) compared to the control group (2.31 ± 1.17%, 4.13 ± 0.78%, and 6.85 ± 1.01, respectively). In addition, there was a significant Os% and RM% difference between different post-implantation times in UiO-66 implanted group.

The Col% was significantly higher in UiO-66 implanted group than control group at post-implantation 4, 8, and 12 weeks with the percentages of 17.40 ± 1.02%, 30.05 ± 2.61%, and 31.61 ± 1.91%, compared to the control group 0.85 ± 0.21%, 1.17 ± 0.19%, and 2.02 ± 0.43%, respectively. Additionally, the Col% was significantly lower on week 4 than on weeks 8 and 12.

### IHC examination

The UiO-66 implanted group showed CD34^+^ mesenchymal stem cells surrounding the UiO-66 material and arranged in a meshwork of interconnected cells (Fig. supp 1).

### qRT-PCR analysis

**Fig. 10:**
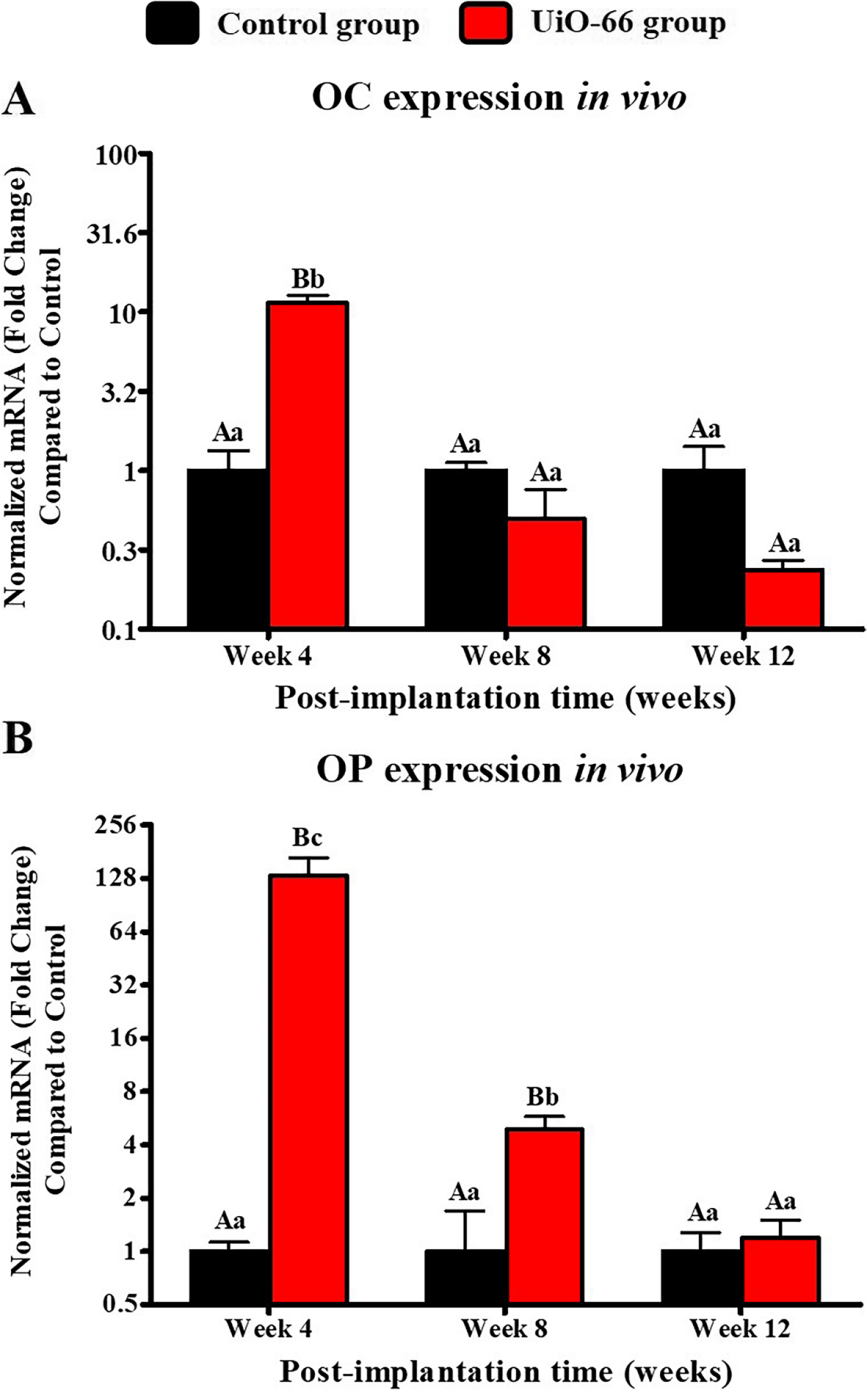
In vivo qRT-PCR analysis. qPCR analysis for mRNA expression of osteocalcin (OC) (A) and osteopontin (OP) (B) in control and UiO-66 implanted groups at weeks 4,8, and 12 post-implantations. Error bars represent means ± SD; *n* = 3 for each group and time point. Bars with the same letter represent values that are not significantly different (two-way ANOVA followed by Tukey's HSD post hoc test). A, B, and C: significance between groups; a, b, and c: significance between time points

The OC and OP expression levels in UiO-66 implanted group showed a decreased manner from week 4 to week 12 after implantation, where their expression levels decreased greatly after post-implantation week 4 (Fig. 10). On week 4 after surgery, OC and OP expressions were higher in UiO-66 implanted defects than in control defects, however; OP expression was significantly higher in UiO-66 implanted group compared to that in the control group at week 8 post-implantation. Additionally, the UiO-66 implanted defects and control ones showed insignificant levels of OC and OP between each other at week 12.

Significant OC and OP expression levels were reported between week 4 and both weeks 8 and 12 after implantation in UiO-66 implanted group. Furthermore, OP expression level was higher in week 8 compared to week 12.

## Discussion

Non-healed critical-sized bone defects remain a challenging problem in both human and veterinary orthopedics as these defects have no tendency to heal spontaneously without additional intervention [3]. Recently, the application of bone tissue engineering has been employed to hasten the osteo-regeneration process [5, 9]. The ideal synthetic bone grafting material should be economic, easily applied, effective in simulating the osteoconductive and osteoinductive extracellular matrix microenvironment, and has excellent mechanical properties. Additionally, the material should be biocompatible with the capability to support cell attachment and growth [1, 33]. In this study, the potential of UiO-66 nanomaterial to accelerate bone healing was investigated in vitro and in vivo in a rabbit femoral bone defect model.

The UiO-66 nanomaterial was prepared by the solvothermal reaction of terephthalic acid and zirconium using DMF as a solvent and acetic acid as a modulator. In this reaction, terephthalic acid coordinates to zirconium clusters at 120 ^o^ C leading to a white precipitate of crystal of UiO-66 [13]. The XRD pattern of synthesized and simulated UiO-66 was in good matching, indicating its high purity [13, 24, 30].

In bone tissue engineering, the cytocompatibility of the biofabricated materials is an essential characteristic feature that should be investigated to emphasize their potential for supporting bone cell growth and attachment in vivo [34]. Consequently, the material’s cytotoxicity was explored through culturing of hFOB cells using extracts of UiO-66 nanomaterials. MTT and Live/ Dead assays revealed that UiO-66 was a cytocompatible nanomaterial. These findings concurred with Aghajanzadeh et al*.* [35] who reported a normal cell growth upon culturing human skin fibroblasts on UiO-66 for 48 h.

The interaction of bone graft substitutes with blood is another critical factor influencing hemocompatibility since the interaction of blood with grafts is pivotal for tissue integration [36]. Therefore, we have conducted a hemolysis screening test to assure the hemocompatibility of the designated nanomaterial. Our results displayed a non-significant rate of hemolysis when UiO-66 nanomaterial contacted RBCs compared to the negative control group. These findings confirmed that UiO-66 is a hemocompatible nanomaterial as reported previously [37]. These data suggested that UiO-66 nanomaterial might have no impairing effect on the normal growth of osteoblasts and blood cells; subsequently, it’s in vivo implantation would not elicit changes in the normal physiological microenvironment.

Numerous collagenous and non-collagenous proteins were expressed in the process of bone repair such as Col-I, OC, and OP. These proteins play a crucial role in osteoblast proliferation and differentiation, bone mineralization, and bone remodeling [33, 38]. Thus, we quantitively investigated the expression of Col-I, OC, and OP proteins using qPCR to assess the potential of UiO-66 nanomaterial to stimulate the osteogenic activity. Our results showed a significant upregulation of the osteoblast marker genes upon culturing of hFOB on UiO-66 nanomaterials. These findings suggested that the fabricated UiO-66 nanomaterial is able to induce and support osteoblast functions in vitro, as described in previous studies [32, 39]. These upregulated expressions of Col-I, OC, and OP may result from the interaction of Runx2 with the promotor regions of osteoblast-specific genes. This interaction is suggested to be initiated by a contact interface between Zr ions of UiO-66 nanomaterial and Runx2 [32, 38, 40]. In addition, the highest OC and OP expressions were noticed on day 14, while their levels abruptly declined on day 21. These observations might be related to the complete mineralization of cells as reported by previous studies [39, 41, 42].

To investigate the in vivo bone-forming potential of the UiO-66 nanomaterial, the designed nanomaterial was implanted in a critical-sized bone defect model (5 mm in diameter and 10 mm in depth) in a rabbit’s femoral condyle [43]. The cellular response of host immunity against the implanted biomaterials regulates and induces the biomaterial-mediated osteogenesis cascade. These reactions against the implanted biomaterials elicit the inflammatory cells influx and fibrosis of the surrounding tissues. The minimal host inflammatory reaction against the implanted material is essential for maintaining the biological effects and functions of the implanted material and indicating its biocompatibility [44, 45]. Our results revealed a mild inflammatory cell infiltration in the UiO-66 implanted defects, proving their excellent biocompatibility and providing a suitable milieu for induction of the stages of osteogenesis. The influx of inflammatory cells, particularly macrophages, in the defect area is a key criterion for biodegradation of implanted material [46]. The implanted UiO-66 nanomaterial in this study displayed gradual biodegradation in a time-dependent manner.

Moreover, the inflammatory cells, mainly macrophages, secrete osteoinductive growth factors as bone morphogenetic proteins (BMPs), vascular endothelial growth factor (VEGF), and transforming growth factor β (TGF-β), and stimulate several cytokines including tumor necrosis factor-α (TNF-α), interleukin 1-β (IL1-β), IL-6, and IL-10. These cytokines and growth factors promote the process of osteogenesis via recruitment and migration of the undifferentiated MSCs with osteogenic potential to the implanted defect area from the bone marrow and peripheral blood and ultimately differentiation, osteoblast maturation, collagen organization, and mineralization [44, 45, 47, 48]. The undifferentiated MSCs play a critical role in bone regeneration and remodeling as they aggregate and migrate to the central zones of the defect through the disintegrated UiO-66 material creating a proper microenvironment that stimulates their differentiation into osteoblasts and activates the secretion of various growth factors that enhance the osteogenesis process [49–53]. Consequently, osteogenesis was boosted in UiO-66 implanted group, whereas MSCs migrated, proliferated, and differentiated to osteogenic cells then to bone-forming osteoblast cells. Osteoblast cells are responsible for collagen deposition; then they are transformed into osteocytes. At last, the newly formed woven bone was remodeled into lamellar bone [54, 55].

Our findings revealed the presence of CD34^+^ cells surrounding the disintegrated UiO-66 material. CD34 has been regarded as a MSCs marker with the in vitro potential of CD34^+^ expressing cells to differentiate into osteoblast cells [50, 56, 57]. Additionally, the implanted group showed the formation of woven immature bone throughout the defect, which eventually remodeled into the lamellar bone in the periphery of the implanted defects. The ability of UiO-66 scaffolds to induce bone formation might be contributed to BMP-2/SMAD signaling pathway stimulation, which induces osteoblasts differentiation and function through promoting Runx2 and Osterix (Osx) production that enhance the functions of mature osteoblasts via stimulation of all major osteoblast-related genes required for collagen deposition and mineralization [32, 58, 59].

Importantly, the ability of UiO-66 scaffold nanomaterial to stimulate in vivo osteogenesis was confirmed by investigating the OC and OP gene expression levels. Our results revealed that UiO-66 implanted group could upregulate these osteogenic genes. This might be attributed to the capability of UiO-66 nanomaterial to enhance the BMP/SMAD signaling pathway. In this pathway, UiO-66 increase BMP-2 secretion that binds to BMP receptors I and II, stimulating the binding of Smad-1/5 with Smad-4, which increases the production of transcription factors as Runx2 and Osx. These transcription factors increase the expression of the osteogenic markers, including OC and OP [32, 60]. Regarding the downregulation of OC and OP expressions levels in the UiO-66 implanted group at post-implantation 8 and 12 weeks, this might be correlated to the normal reduction in the synthesis of bone matrix proteins in the late stages of bone healing as reported by Honma et al*.* [61] and Itagaki et al*.* [62] who investigated the osteoblasts and osteocytes potential for bone matrix proteins production during bone formation and reported the significant increase in bone matrix proteins production toward the 2^nd^ week with the highest level was on the 4^th^ week and then decreased. Furthermore, the overexpression of OP on week 4 after implantation may be associated with its secretion by both macrophages and osteoblast lineage cells [63].

In this study, it is worth mentioning that there was a limitation in assessing the UiO-66 implanted defects radiographically because UiO-66 nanomaterial displays a higher radiopacity than normal bone due to the higher atomic number of Zr than calcium. Therefore, it could mask the process of bone formation in the defect areas [64].

## Conclusion

The UiO-66 scaffold is a cytocompatible material that supports bone cell growth and attachment and upregulates osteoblast marker genes in vitro. In addition, it has an in vivo behavior of partial biodegradation of the nanomaterials and replacement by newly formed bone tissue, indicating its potential to induce osteogenesis. To the best of our knowledge, this is the first study to report the UiO-66 nanomaterial as a promising biomaterial that could be used to fabricate scaffolds for the reconstruction of critical-sized bone defects in rabbit femoral condyle model. However, UiO-66 biodegradability and biomechanical properties should be further studied in future research. In addition, future studies on a higher number of animals comparing the healing process to other well-established scaffolds such as tricalcium phosphate and demineralized bone matrix are required.

## Materials and methods

### Study design

**Fig. 11:**
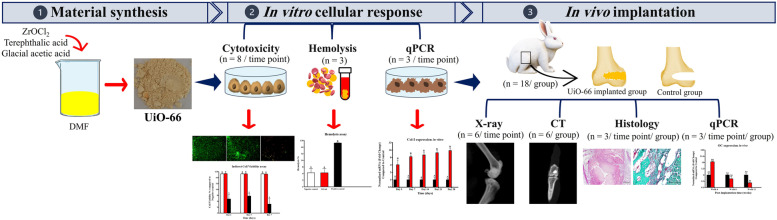
Summary of the study design

The present study was carried out in three main stages as summarized in Fig. 11, including material synthesis and characterization, in vitro experiments, and in vivo implantation of UiO-66 nanomaterial in rabbit femoral condyle bone defects.

### Ethics approval

The study protocols were approved by the Institutional Animal Care and Use Committee of Research Facilities, Faculty of Veterinary Medicine, Assiut University, Egypt in accordance with the Animal Research: Reporting of In Vivo Experiments (ARRIVE) guidelines. All methods were performed in compliance with the relevant guidelines and regulations e.g., Egyptian bylaws for animal use, OIE animal welfare standards, Directive 2010/63/EU in Europe, and American Veterinary Medical Association (AVMA) Guidelines for the Euthanasia of Animals (2020).

### Chemicals

Terephthalic acid, zirconium oxide chloride (ZrOCl_2)_, acetic acid, and dimethylformamide (DMF) were purchased from Sigma Aldrich (Germany). All the chemical reagents were used without further purification.

### Synthesis and characterization of UiO-66 nanomaterial

UiO-66 was prepared according to our previous reference [13]. A mixture of ZrOCl_2_ (750 mg), terephthalic acid (740 mg), and glacial acetic acid (4 mL) was dissolved in DMF (90 mL). The mixture was dispersed via ultrasonication for 30 min. The solution was transferred to a Teflon-lined stainless-steel autoclave (100 mL) and heated at 120 ºC for 24 h. The product was washed three times with DMF (3 × 30 mL), and ethanol (3 × 30 mL) before drying overnight in an oven at 100 ºC.

The phase purity of the prepared material was characterized using powder x-ray diffraction (XRD; Philips 1700 diffractometer, Germany) with a Cu–Kα radiation diffractometer. The morphology and size of these nanomaterials were studied using a transmission electron microscope (TEM; JEM-2100; JEOL, Japan).

### Indirect contact cytotoxicity assay

Extracts were prepared from the UiO-66 nanomaterial scaffolds to evaluate the potential cytotoxic risk as described previously [34, 65]. The extracts of the UiO-66 scaffold were prepared by incubating samples (after ethylene oxide gas sterilization) in the serum-free 1: 1 mixture of Ham’s F12 and Dulbecco Modified Eagle’s minimal essential medium (DMEM) supplemented with 1% penicillin/streptomycin (p/s, Gibco, USA) culture medium under the condition of 37 °C/120 r/minutes for 72 h, according to a ratio standard of 0.2 g/mL of culture medium [46]. The supernatant was obtained and centrifuged to prepare the conditioned extracts and filtered using 0.4 μm filters, then stored at 4 °C before the cytotoxicity test.

Human fetal-osteoblast cell line (hFOB 1.19; American Type Culture Collection (ATCC), USA) were cultured in a 1:1 mixture of Ham’s F12 and DMEM supplemented with 10% fetal bovine serum (FBS; Hyclone, USA), 2.5 mM L-glutamine (Gibco, USA), and 1% p/s in a humidified incubator at 34 °C and 5% CO_2_. After reaching the full confluency, the cells were trypsinized and placed at a density of 15 × 10^3^ in a 48-well plate for 24 h using the complete culture medium. Then, the medium was aspirated, followed by the addition of 500 µL conditioned or control medium after adding 10% FBS. In the negative control, cells were cultured with a complete medium only, while in positive control wells, the cells were cultured in the presence of 20% dimethyl sulfoxide (DMSO). The cell response against the extracts was investigated by measuring the metabolic activity of cells using [3-(4,5-dimethylthiazol)-2-yl]-2,5- diphenyltetrazolium bromide (MTT) assay after 1, 3, and 7 days of culture. Briefly, 50 μL of MTT solution (5 mg /mL; Sigma-Aldrich, St Louis, MO, USA) was added to each well and incubated at 37 °C for 4 h. Then, the MTT-containing media was aspirated, and 250 μL DMSO was added to solubilize the formazan crystals. After incubation for 10 min, 100 μL aliquots from the wells were pipetted into another 96-well plate. The intensity of the colored product was quantitatively measured at a wavelength of 570 nm using a spectrophotometer. The cell viability was expressed as the percentage of activity expressed by treated cells as compared to the negative control.

For qualitative assessment of the cell viability, staining with a Live/Dead assay kit (calcein-AM/ethidium bromide homodimer, Invitrogen) after 7 days of culture according to the manufacturer’s instructions and imaged using a fluorescence microscope (Olympus, Tokyo, Japan).

### Hemolysis assay

The hemolysis that may occur due to the designed nanomaterial was measured to evaluate the material hemocompatibility as previously described [66]. Briefly, fresh blood was collected from healthy live dogs and directly transferred to the laboratory. Erythrocytes were separated by centrifuging at 2,000 g for 15 min, followed by dilution in 1 × PBS to create an erythrocyte suspension with 2 × 10^9^ cells/mL. Sample of each nanomaterial was placed in a glass tube containing 5 mL of erythrocyte suspension and kept at room temperature on a shaker with 125 rpm for 60 min. Erythrocyte suspension without any specimens was utilized as a negative control, whereas tubes containing 25 mg of sodium dodecyl sulfate (SDS) added to the erythrocytes were used as positive controls. Afterward, 1 mL of the suspension was collected from each tube and centrifuged for 3 min at 3,000 g. Finally, the absorbance of the supernatant was examined at a wavelength of 545 nm using a spectrophotometer, and hemolysis percentage was calculated as in the equation:

$$\mathrm{Hemolysis}\;\left(\%\right)=\left[\left(\mathrm S545-\mathrm N545\right)\div\left(\mathrm P545-\mathrm N545\right)\right]\times100,$$

Where the absorbance for samples, negative control, and positive control were represented by S545, N545 and P545, respectively.

### Quantitative Polymerase Chain Reaction (qPCR) analysis

The UiO-66 nanomaterial was placed into 48-well plates to assess its ability to support the attachment of hFOB 1.19 cells. Briefly, 500 μL of media containing 10 × 10^3^ cells were added to the surface of the disks, and the plate was incubated at 34 °C in 5% CO_2_ for 28 days. After 3, 7, 14, 21, and 28 days of culture, qPCR analysis was performed as explained previously [67] for the primer sequences listed in Table 1. At the different time points, total RNA was isolated from the harvested cells and transcribed into cDNA using the NucleoSpin RNA Mini kit (Macherey–Nagel GmbH & Co., Germany) and TOPscrip RT DryMIX (Enzynomics, South Korea), respectively. qPCR was performed using TOPreal qPCR 2 × PreMIX (Enzynomics) on a StepOnePlus real-time PCR system (Thermo Fisher Scientific) following the manufacturer’s recommendations. Analysis of the relative gene expression was calculated by the 2^−ΔΔCt^ method with glyceraldehyde 3-phosphate dehydrogenase (GAPDH) as the internal control.

**Table 1:** Primer sequences used for qPCR analysis of in vitro and in vivo osteogenic gene expressions

Primer	Primer Sequences	
	**Forward**	**Reverse**
**Human Collagen-I**	5’- CAG CCG CTT CAC CTA CAG C -3’	5’- TTT TGT ATT CAA TCA CTG TCT TGC C -3’
**Human Osteocalcin**	5’- ACA CTC CTC GCC CTA TTG -3’	5’- GAT GTG GTC AGC CAA CTC -3’
**Human Osteopontin**	5’- CTC AGG CCA GTT GCA GCC -3’	5’- CAA AAG CAA ATC ACT GCA ATT CTC -3’
**Human GAPDH**	5’-ACA GTC AGC CGC ATC TTC TT-3’	5’-GAC AAG CTT CCC GTT CTC AG-3’
**Rabbit Osteopontin**	5’-GCTCGATGGCTAGCTTGTCT-3’	5’-ACAATATAAGCGCGAGGCCA-3’
**Rabbit Osteocalcin**	5’-GTTCCCTTCCTCCTTGATTT-3’	5’-TCTACCAGTTGCAGCCTGAC-3’
**Rabbit Beta-actin**	5-’CAGGAAGGAGGGCTGGAACA-3’	5’-ATCGTGCGGGACATCAAGGA-3’

### Critical-sized bone defect model and implantation procedure

A total of 36 mature six-month-old male New Zealand white rabbits weighing 2.5 to 3.0 kg were used for this experiment to evaluate the potential of the UiO-66 scaffold in enhancing the repair of the critical-sized bone defect. Rabbits were checked clinically and radiographically to confirm their skeletal maturity and normal bone structure, and then were housed individually in stainless-steel boxes with accessible commercial rabbit chow and water. All animals were allowed to accommodate in their cages for 14 days before surgery. Animals were divided randomly into; control group (empty defects, *n* = 18) and UiO-66 group (UiO-66 implanted defects, *n* = 18).

All surgical procedures were carried out under strict aseptic preparations. Animals were fasted for 8 h before surgical operations. They were subjected to surgery under the effect of general anesthesia induced by a mixture of xylazine HCl (3 mg/kg, Xyla-ject 20% ®: ADWIA Co., Egypt) and ketamine HCl (40 mg/kg, Ketamine 50% ®: Sigma-Tec, Egypt), and maintained with oxygen (2 L/ minute) and isoflurane (2.5–3%, Forane ®: AbbVie, England). After induction of general anesthesia, the right hindlimb was shaved and disinfected and the animal was placed in lateral recumbency during surgery. Critical-sized bone defects were created in the middle region of the lateral femoral condyle (5 mm in diameter,10 mm in depth) using dental micromotor with low-speed contra (Strong, Korea) and trephine burrs as described previously [43]. In brief, a longitudinal 5 cm skin incision proximally from the patella was performed parallel to the lateral femoral condyle. The underlying subcutaneous tissue and muscles were dissected parallel to the skin incision and the femoral shaft. Then, the periosteum was reflected, and lateral femoral condyles were exposed. The defects were created under continuous saline irrigation to minimize thermal injury and bone damage. To create these confined critical-sized bone defects, a trephine burr (Ø = 3 mm; Osung, Korea) was applied on the lateral femoral condyles perpendicular to the longitudinal femoral axis. Then, the defect was enlarged using a larger trephine burr (Ø = 5 mm; Oxy, Italy) to reach a confined bone defect size (Ø 5 × 10 mm). The defects were thoroughly washed with physiological saline and dried with sterile gauze to remove bone debris. The defects were either filled with UiO-66 material or left empty as control one. The muscle attachment, subcutaneous tissue, and skin were sutured in a routine manner. During the consecutive postoperative 5 days, each rabbit was given a subcutaneous injection of penicillin (40 mg/kg, Pen & Strep ®: Norbook, Egypt) to prevent infection, in addition to meloxicam (0.6 mg/kg, Mobitil ®: MUP, Egypt) to suppress pain. Animals were allowed to move freely in their boxes without restrictions. At 4, 8, and 12 weeks after surgery, six rabbits from each group were sacrificed at each time for assessment of bone regeneration.

### Clinical investigation

All animals were examined daily for postoperative complications and any signs of illness including infection, fracture, activity level, gait, and mobility of the hindlimbs.

### Radiographic evaluation

Latero-medial (LM) radiographs were acquired to determine the newly formed bone in the defect regions. The rabbit femurs were radiographed just after surgery and at different evaluation times using a fixed x-ray apparatus (50 kV and 10 mA/s; Philips Super 80 CP, Germany) and an ultra-high-definition film.

### Computed tomography (CT) examination

CT scanning of the bone defects was performed at week 12 after surgery using Philips 128 slice scanner CT apparatus (120 kV and 53 mA/s, 1 mm thickness; Germany) intending to assess the bone repair at the defect areas.

### Gross evaluation of the femoral condyle bone defects

The femoral condyles were resected with the removal of all soft tissues at weeks 4, 8, and 12 after the operation. The condyles were assessed grossly for the signs of infection, inflammation, grafting material incorporation, and new bone formation.

### Histological assessment

At different evaluation times, femoral condyles (*n* = 3 from each group at each time point) were harvested and fixed in neutral buffered formaldehyde (10%, pH 7.2), and subsequently, samples were decalcified using formic acid 25% (25 mL formic acid, 75 mL distilled water, and 5 mL 40% formaldehyde) at a pH of 7 and 37 °C for 4 weeks.

Formalin-fixed bone samples were routinely dehydrated in ascending grades of ethanol, cleared in methyl benzoate, and then embedded in paraffin wax. Paraffin sections at 5-µm in thickness were cut perpendicular to the longitudinal axis of the femur and finally stained with Hematoxylin and Eosin (H&E) for general histological examination of the defects including signs of bone regeneration, neovascularization, cellular constituents, and the remaining implanted material [68], in addition to Periodic acid Schiff (PAS) staining for glycosaminoglycans observation [69]. The paraffin sections were observed by an Olympus BX51 microscope (Japan), and the images were taken by an Olympus DP72 camera (Japan) adapted into the microscope.

### Histochemical staining of bone collagen

Crossmon's trichrome and Sirus red stains were a valuable method used for assessment of the newly formed bone collagen content, whereas Crossmon's trichrome staining was used to examine the bone collagen fibers deposition within the defect area and Sirus red staining was used to distinguish mature bone collagen from immature bone collagen [68, 70].

The paraffin-embedded sections were deparaffinized, rehydrated, and stained with Crossmon's trichrome stain and Sirus red stain according to the manufacturer's protocol. Afterward, slides were dehydrated, made transparent with xylene, and mounted. At last, stained sections were examined under microscope and photographed using a digital camera.

### Histomorphometric analysis

Quantitative measurements were conducted to determine the percentages of osteoid tissue (Os%) and remaining material (RM%) areas in the H&E-stained slides (*n* = 3 slides for each defect). In addition, the percentage of mature bone collagen area (Col%) was calculated in Siris red-stained sections (*n* = 3 slides for each defect). Using threshold area fraction, the areas of osteoid tissue, the remaining material, and mature bone collagen were detected in the region of interest (ROI, the entire defect area, 25.6mm^2^) using ImageJ software (National Institutes of Health, Bethesda, MD, USA) [43, 71].

The Os%, RM%, and Col% were described as a percentage of the total area of the defect, expressed as mean ± SD, and calculated as follows:

$$Os\%=\frac{Os}{ROI}\times100,\;RM\%=\frac{RM}{ROI}\times100,\;Col\%=\frac{Col}{ROI}\times100$$

where Os, RM, and Col were used to indicate the areas of osteoid tissue, residual material, and mature collagen, respectively.

### Immunohistochemical (IHC) examination

IHC of CD34 was carried out at week 12 after surgery according to Abd-Elkareem [72] using CD34 monoclonal antibody (Catalog Number: CM 084 A, B, C, Biocare Medical, USA) and ultravision one detection system HRP polymer & AEC chromogen (Catalog Number: TL-015-HAJ, Thermo Fisher Scientific, USA).

### Quantitative real-time PCR (qRT-PCR) analysis

After sacrificing the animals at 4,8, and 12 weeks after surgery, 3 samples were carefully obtained from each group at each evaluation time for qRT-PCR analysis. Soft tissues and periosteum were stripped off and samples were collected under complete aseptic preparations. The collected samples were directly immersed in a nontoxic tissue storage reagent (RNAlater solution, Thermo Scientific, USA) and then samples were stored at -80 °C for further steps.

Afterward, RNA extraction was carried out by frozen samples grinding using a mortar under liquid nitrogen. Total RNA was extracted from the bone samples using the TRIzol® Reagent (life technologies) according to the manufacturer's protocol. Subsequently, reverse transcription and qRT-PCR were proceeded for OC and OP as described previously for in vitro qPCR. Forward and reverse primers are listed in Table 1.

### Statistical analysis

The obtained data were statistically analyzed with a statistical software program (IBM SPSS version 21). Data are presented as mean ± standard deviation (SD) with a significance at p < 0.05. One-way analysis of variance (ANOVA) followed by Tukey's post hoc test was used to analyze the results of cytotoxicity assay (*n* = 8), hemocompatibility, in vitro expression of collagen type-I (Col-I), OC, and OP, and in vivo RM% (*n* = 3 for each time). Moreover, two-way analysis of variance (ANOVA) followed by Tukey's post hoc test was performed to analyze the results of Os%, Col % (*n* = 3 for each defect at each time), and in vivo expressions of OC and OP (*n* = 3 for each time).

## Supplementary Information

**Additional file 1: Fig. Supp 1. **Immunohistochemical staining of bone defect in rabbit femoral condyle at week 12 after surgery. The repair site of the femoral condyle in the control (B) and UiO-66 implanted (C) bone defects were stained with CD34 monoclonal antibody. Black arrowheads: CD34^+^ mesenchymal stem cells; red asterisks: implanted UiO-66 nanomaterial. Scale bars = 50 µm.

## Data Availability

The data that support the results of this study are available from the corresponding author on reasonable request.

## Abbreviations

BMPs: Bone morphogenetic proteins
Col: Collagen
Col-I: Collagen type-I
CT: Computed tomography
DMEM: Dulbecco Modified Eagle’s minimal essential medium
DMSO: Dimethyl sulfoxide
FBS: Fetal bovine serum
H&E: Hematoxylin and Eosin
hFOB: Human fetal-osteoblast cell line
IL: Interleukin
LM: Latero-medial
MOFs: Metal–organic frameworks
MSCs: Mesenchymal stem cells
MTT: [3-(4,5-dimethylthiazol)-2-yl]-2,5- diphenyltetrazolium bromide
OC: Osteocalcin
OP: Osteopontin
Os: Osteoid tissue
Osx: Osterix
p/s: penicillin/streptomycin
PAS: Periodic acid Schiff
qRT-PCR: Quantitative real-time Polymerase Chain Reaction
RM: Remaining material
SDS: Sodium dodecyl sulfate
TEM: Transmission electron microscope
TGF-β: Transforming growth factor β
TNF-α: Tumor necrosis factor-α
UiO-66: University Institute of Oslo-66
VEGF: Vascular endothelial growth factor
XRD: X-ray diffraction
Zr: Zirconium
ZrOCl_2_: Zirconium oxide chloride
