# Supplementary figures and images for: Enhancement of critical-sized bone defect regeneration using UiO-66 nanomaterial in rabbit femurs
Source: BMC Vet Res. 2022 Jul 5;18:260. doi: 10.1186/s12917-022-03347-9 (PMC9254639; doi:10.1186/s12917-022-03347-9)

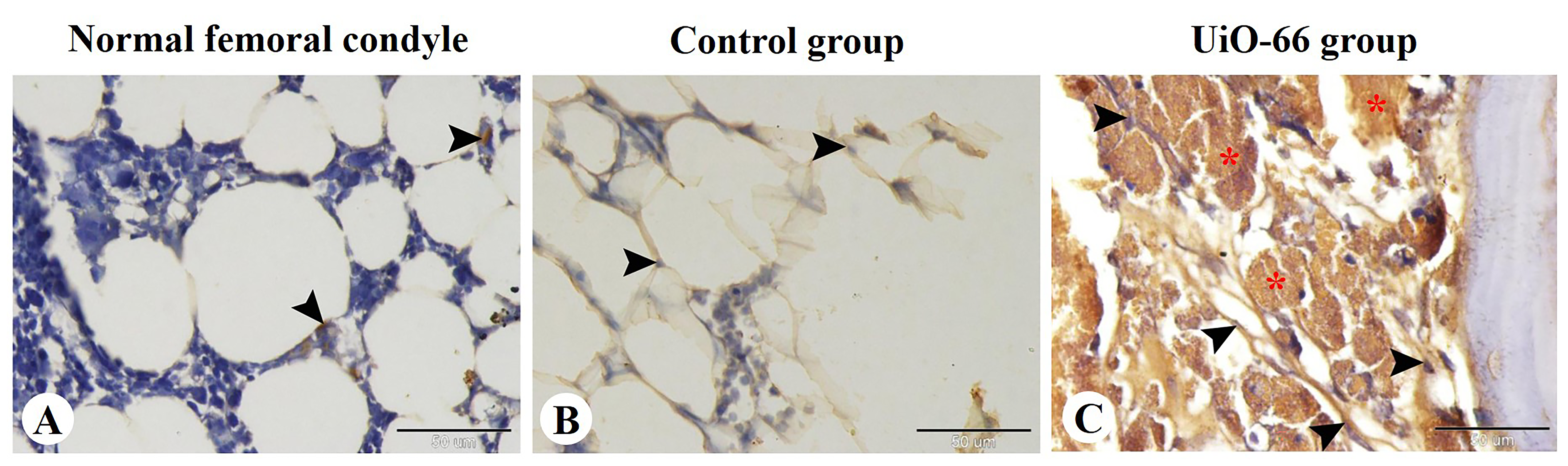

Supplement: Supplementary file 1 — Additional file 1: Fig. Supp 1. Immunohistochemical staining of bone defect in rabbit femoral condyle at week 12 after surgery. The repair site of the femoral condyle in the control (B) and UiO-66 implanted (C) bone defects were stained with CD34 monoclonal antibody. Black arrowheads: CD34+ mesenchymal stem cells; red asterisks: implanted UiO-66 nanomaterial. Scale bars = 50 µm. [file 12917_2022_3347_MOESM1_ESM.tif]
